# The Role of Short Chain Fatty Acids in Inflammation and Body Health

**DOI:** 10.3390/ijms25137379

**Published:** 2024-07-05

**Authors:** Yuhang Du, Changhao He, Yongcheng An, Yan Huang, Huilin Zhang, Wanxin Fu, Menglu Wang, Ziyi Shan, Jiamei Xie, Yang Yang, Baosheng Zhao

**Affiliations:** 1 Department of Pharmacology of Chinese Materia Medica, School of Chinese Materia Medica, Beijing University of Chinese Medicine, Beijing 102488, China; 20230935235@bucm.edu.cn (Y.D.); hechanghaotcm@bucm.edu.cn (C.H.); hyss111@126.com (Y.A.); 18504732059@163.com (H.Z.); wml1251247317@163.com (M.W.); hbxiejiamei@163.com (J.X.); bucmyang2020@163.com (Y.Y.); 2 College of Life Sciences, Beijing University of Chinese Medicine, Beijing 102488, China; huangyan4826@163.com (Y.H.); fwx062@163.com (W.F.); 18645681606@163.com (Z.S.); 3 Beijing Research Institute of Chinese Medicine, Beijing University of Chinese Medicine, Beijing 100029, China

**Keywords:** short chain fatty acids, gut microbiota, inflammation, signaling pathways, metabolic disease

## Abstract

Short chain fatty acids (SCFAs), mainly including acetate, propionate and butyrate, are produced by intestinal bacteria during the fermentation of partially digested and indigestible polysaccharides. SCFAs play an important role in regulating intestinal energy metabolism and maintaining the homeostasis of the intestinal environment and also play an important regulatory role in organs and tissues outside the gut. In recent years, many studies have shown that SCFAs can regulate inflammation and affect host health, and two main signaling mechanisms have also been identified: the activation of G-protein coupled receptors (GPCRs) and inhibition of histone deacetylase (HDAC). In addition, a growing body of evidence highlights the importance of every SCFA in influencing health maintenance and disease development. In this review, we summarized the recent advances concerning the biological properties of SCFAs and their signaling pathways in inflammation and body health. Hopefully, it can provide a systematic theoretical basis for the nutritional prevention and treatment of human diseases.

## 1. Introduction

Short chain fatty acids (SCFAs) are organic acids produced in the intestinal lumen by the bacterial fermentation of undigested dietary carbohydrates, dietary or endogenous proteins, such as mucous and sloughed epithelial cells [1]. Bacteria of the Bacteroidetes phylum are known to produce high levels of acetate and propionate, whereas bacteria of the Bacillota are known to produce high amounts of butyrate [2]. Acetate (C2), propionate (C3) and butyrate (C4) account for more than 95% of the SCFAs in the gut, with an estimated ratio of about 3:1:1 in the gut [3,4]. SCFAs are vital mediators between the microbiota and host physiology. A decreased production of SCFAs is associated with metabolic diseases [5,6]. SCFAs have been identified as important metabolic biomarkers of disease-related changes [7]. They are strongly associated with a variety of diseases, including gastrointestinal disorders, obesity, diabetes, inflammation, kidney disease, cancer and neurological disorders.

**Figure 1:**
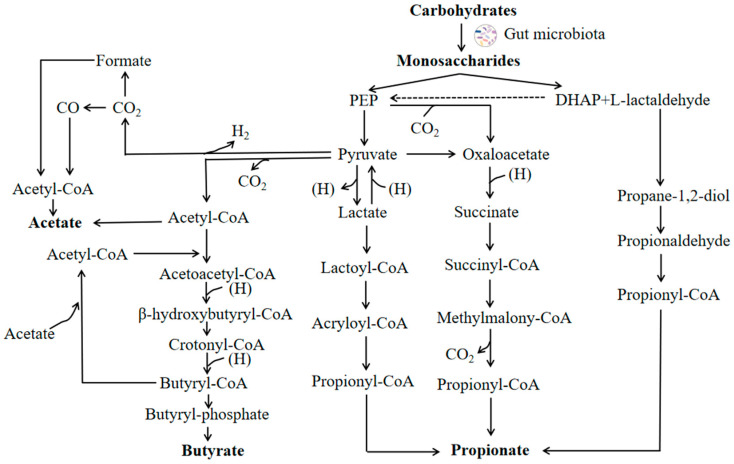
A schematic diagram of carbohydrate fermentation pathways producing acetate, propionate and butyrate. PEP, phosphoenolpyruvate; DHAP, dihydroxyacetonephosphate.

The microbial conversion of dietary fiber to monosaccharides in the gut is a complex process involving a number of principal events (reactions) mediated by the enzymatic repertoire of specific members of the gut microbiota [8]. (Figure 1) The major end products from these fermentations are the SCFAs. One of the major SCFAs, acetate, can be produced directly from acetyl-CoA or via the Wood–Ljungdahl pathway using hydrogen and carbon dioxide or formate. Propionate is produced from the conversion of succinate to methylmalonyl-CoA via the succinate pathway. It can also be synthesized from acrylate with lactate as a precursor via the acrylate pathway [9] and via the propanediol pathway using deoxyhexose sugars (such as fucose and rhamnose) as substrates [10]. Butyrate is formed from the condensation of two molecules of acetyl-CoA and subsequent reduction to butyryl-CoA by phosphotransbutyrylase and butyrate kinase [11] or by the butyryl-CoA:acetate CoA-transferase route [12].

Following their production in the colon, SCFAs are rapidly absorbed by the colonocytes. After supplying colonocytes, the remaining SCFAs are transported through the blood to various parts of the body [13]. Only a small proportion (approximately 5–10%) is excreted in the feces [14]. Acetate produced by bacterial fermentation in the colon enters the bloodstream and is mixed with acetate released by tissues and organs [15]. Up to 70% of the acetate is taken up by the liver, where it is used not only as an energy source but also as a substrate for the synthesis of cholesterol and long chain fatty acids and as a cosubstrate for glutamine and glutamate synthesis [16]. Other tissues, including the heart, adipose tissue, kidney and muscle, metabolize the rest of the acetate [16]. To prevent high SCFAs concentrations in the blood, the liver clears the major part of propionate and butyrate from the portal circulation [16]. Propionate acts as a precursor for gluconeogenesis in the liver [17]. After the conversion of propionate into propionyl-CoA, propionyl-CoA is converted to succinyl-CoA, which enters the tricarboxylic acid (TCA) cycle and is converted to oxaloacetate, the precursor of gluconeogenesis [16]. The majority of butyrate is metabolized by colonocytes, where it is oxidized to ketone bodies and CO_2_; the remainder is oxidized by hepatocytes, preventing toxic systemic concentrations [16]. As discussed above, these SCFAs can be used as substrates to synthesize sugars or lipids and can also be used as cytokines to regulate metabolism (Table 1) [18,19].

The immune system is composed of two distinct branches: adaptive immunity, which develops in response to antigen stimulation and subsequent specific responses to the antigen, and innate immunity, which is relatively nonspecific in nature [20]. SCFAs have become well-recognized as mediators between the microbiota and mucosal immune cell populations, and regulation by SCFAs extends to both the innate and adaptive immune compartments both locally in the gut and systemically [21]. SCFAs can participate in intestinal immune homeostasis by regulating T-cell polarization and inducing T-cell differentiation into effector and regulatory T cells (Tregs) [22]. SCFAs exert inhibitory and accelerating effects on neutrophils and influence the immunoregulation of monocytes and macrophages [22]. Zheng et al. (2024) demonstrated that the potential mechanisms for the improvement of dextran sodium sulfate (DSS)-induced colitis include alterations in the diversity and composition of the gut microbiota, as well as the upregulation of SCFA levels and Treg production [23]. Furthermore, a growing body of evidence indicates that each SCFA plays a pivotal role in influencing health maintenance and disease development.

Therefore, it is critical to understand exactly how SCFAs affect body energy homeostasis to improve the clinical efficacy of microbiota regulation on metabolic health. In this review, we summarized the biosynthesis, distribution and physiology of SCFAs and discussed their roles in diseases, with the aim of providing a theoretical basis and reference for the treatment of diseases based on SCFA regulation.

**Table 1:** The impact of SCFAs on the basic biochemical pathways in the human body.

SCFAs	Effects on Basic Biochemical Pathways	Refs
acetate	cholesterol and long chain fatty acid synthesis ↑; glutamine and glutamate synthesis ↑; secretion of leptin in adipocytes ↑; fat oxidation ↑	[16,24]
propionate	gluconeogenesis in the intestine and liver ↑; fatty acid synthesis ↓; TC ↓	[8,15,16,25]
butyrate	glycolysis ↓; intestinal gluconeogenesis ↑; liver gluconeogenesis ↓; liver fatty acid oxidation ↑; lipid oxidation and glucose uptake in muscle ↑; lipolysis in adipose tissue ↓	[15,25,26]

## 2. Physiological Mechanisms of SCFAs

Previous studies suggest that SCFAs have two major mechanisms of action: activation of G-protein coupled receptors (GPCRs) and inhibition of histone deacetylase (HDAC) [27].

### 2.1. Ligands for GPCRs

SCFAs can activate the G-protein-coupled receptors GPR41, GPR43 and GPR109A [28,29]. These receptors are also called free fatty acid receptors (FFARs) since they sense free fatty acids [8]. They have attracted considerable interest and are considered promising therapeutic targets for the treatment of metabolic disorders, including diabetes and obesity, as well as for the regulation of inflammatory processes [30,31,32] (Table 2).

#### 2.1.1. GPR43/FFAR2

GPR43, also known as FFAR2, is expressed in white adipocytes, enteroendocrine L cells, intestinal epithelial cells (IECs), pancreatic β-cells, colonic Tregs, M2 macrophages, neutrophils, eosinophils and mast cells [24] and plays an important role in hormone secretion, lipid metabolism, immunomodulation and the nervous system. It is primarily activated by acetate and propionate, followed by butyrate [37]. SCFAs are primarily natural agonists of FFAR2, and previous conformational studies have shown that FFAR2 exhibits a preference for shorter SCFAs [22]. The potencies of the individual SCFAs in activating FFAR2 in humans are ordered as C2 = C3 > C4 > C5 = C1 [13], whereas those for the mouse receptor are ordered as C2 > C3 > C4 [31]. The observed differences in the responses of human and mouse receptors to SCFAs may be attributed to the fact that the mouse receptor shares approximately 84% amino acid sequence similarity with the human receptor [38]. GPR43 exhibits dual-coupling through the G_i/o_ and pertussis toxin-insensitive G_q_ protein families [33]. Stimulation of GPR43 by SCFAs inhibits adenylate cyclase, thereby reducing the production of cAMP from ATP [37]; activates the extracellular signal-regulated kinase (ERK) cascade via interactions with the G_i/o_ family of G proteins; increases intracellular Ca^2+^ levels; and promotes activation of the mitogen-activated protein kinase (MAPK) cascade via interactions with the G_q_ family of G proteins [37]. However, the physiological significance of this dual-coupled signaling mechanism through FFAR2 remains unclear [22]. In addition, β-arrestins are critical regulator and transducer proteins for GPCRs [39]. Lee et al. (2011) demonstrated that GPR43 is associated with β-arrestins, particularly β-arrestin2 (βarr2), and further showed that GPR43 negatively regulates inflammatory cytokines by modulating nuclear factor-κB (NF-κB) activity through βarr2 [40]. In adipose tissues, GPR43 may be involved in regulating obesity and energy accumulation. GPR43 can be expressed on the colonic epithelium, and SCFAs affect several functions of these cells, including proliferation and epithelial barrier function. Stimulation of GPR43 by SCFAs is necessary for the normal resolution of certain inflammatory responses [41]. GPR43-deficient (*Gpr43*^−/−^) mice showed exacerbated or unresolving inflammation in models of colitis, arthritis and asthma [41].

GPR43 is mainly expressed on leukocyte populations, particularly neutrophils [33]. Previous studies have indicated that GPR43 is expressed extensively in immune tissues such as the spleen and in immune cells such as neutrophils [28,42], indicating that SCFAs play an important role in immune responses. GPR43 is a functional receptor for the effects of SCFAs on neutrophils and is highly specifically expressed in neutrophils [33]. The average concentrations of propionate and butyrate in the blood are insufficient to activate GPR41 or GPR43, but the blood concentrations reached by acetate are well within the active range for GPR43 [33]. Using *Gpr43^−/−^* mice, KM et al. (2020) found that although T- and B-cell numbers as well as blood neutrophil numbers were in the normal range, acetate induced a robust calcium flux in mouse neutrophils and in human neutrophils and eosinophils, which was absent in neutrophils from *Gpr43^−/−^* mice, suggesting that GPR43 is the only functional receptor for SCFAs on neutrophils [41]. In a chronic model of DSS-induced colitis, mice fed 200 mM acetate in their drinking water showed a substantial decrease in inflammation, including an increased colon length, a reduced DAI, reduced inflammatory infiltrate and less tissue damage, when compared to wild-type mice not fed acetate, indicating decreased neutrophil infiltration/activation [41].

#### 2.1.2. GPR41/FFAR3

GPR41, also known as FFAR3, is specifically expressed in sympathetic ganglion cells [18]. It can also be expressed in enteroendocrine L cells, enteroendocrine K cells, white adipocytes, myeloid dendritic cells (DCs), the thymus and pancreatic β-cells [22]. In 2003, GPR41 was deorphanized and identified as a receptor for SCFAs [28,33]. It is mainly activated by propionate and butyrate [43]. GPR41 shows a stronger response to the longer SCFAs than FFAR2 [28,33]. The potencies of the individual SCFAs in activating FFAR3 in humans are ordered as C3 = C4 > C5 > C2 > C1 [13,31]. GPR41 is only conjugated by the pertussis toxin-sensitive G_i/o_ families [33]. The stimulation of GPR41 by SCFAs has been demonstrated to inhibit cAMP production and to promote ERK1/2 phosphorylation [22].

GPR41 is expressed in immune cells, adipose tissue, intestine and peripheral nervous system and is involved in energy regulation in response to SCFAs produced from the gut microbiota, which may be beneficial in the treatment of inflammation and metabolic diseases such as obesity and diabetes [43]. It was reported that the propionate-mediated regulation of airway inflammation did not involve GPR43 [44]. The activation of both the GPR41 and GPR43 receptors results in L-cell secretion of PYY and GLP1, which affect various tissues including cardiovascular, pancreatic and brain tissues [39,40]. Furthermore, islets are known to express the receptors GPR41 and GPR43, indicating that SCFAs may be involved in islet-cell metabolism and mitochondrial function [45,46].

#### 2.1.3. GPR109A/HCA2

GPR109A, also known as HCA2, was first identified as a receptor for niacin and is also activated by β-hydroxybutyrate and butyrate [36]. In contrast to GPR41 and GPR43, GPR109A is only activated by butyrate and not by acetate and propionate [47]. While the mouse genome has only one gene for Gpr109 (called GPR109A), the human genome contains two, GPR109A and GPR109B [35]. GPR109B originated from duplication of GPR109A, and thus, these genes are highly similar [36]. GPR109A and GPR109B show 96% identity at the protein level; however, GPR109B does not bind to any SCFAs [47]. GPR109A is a coupled G_i/o_ protein and is expressed in colonic epithelial cells [22] and adipocytes of white and brown adipose tissue, and it is expressed to a lesser extent in keratinocytes, retinal-pigmented epithelium and immune cells, including dermal DCs, monocytes, macrophages and neutrophils [34,35]. As a ligand for GPR109A, butyrate has been demonstrated to reduce intestinal inflammation and to promote the integrity of the intestinal epithelial barrier [48]. In the immune system, GPR43 and GPR109A are expressed on neutrophils, macrophages and DCs, suggesting the roles of SCFAs in immune responses [34,49].

### 2.2. HDAC Inhibitors

Studies have shown that HDAC enzymes are involved in maintaining microbiome-dependent intestinal homeostasis; in particular, HDAC3, a class I histone deacetylase that is highly expressed in the intestinal epithelium, is sensitive to microbial signaling [21]. SCFAs inhibit HDACs, often resulting in increased histone acetylation at direct targets [21]. HDAC inhibitors are widely used in cancer therapy and have also been reported to have anti-inflammatory or immune-suppressive functions [8]. Butyrate and, to a lesser extent, propionate are known to act as HDAC inhibitors, with butyrate being the most potent and widely studied [50]. Although acetate is not traditionally considered to be an HDAC inhibitor, it has been found to inhibit HDAC in activated T cells [51]. As the most effective HDAC inhibitor, the inhibitory efficiency of butyrate on HDAC1/2 can reach about 80% [52]. Propionate, on the other hand, has a maximum inhibition efficiency of about 60% [52]. It has been reported that butyrate and propionate can impact the balance between pro-inflammatory and anti-inflammatory mechanisms by promoting the production of peripheral Tregs [53]. This helps to maintain intestinal immune homeostasis. Gut microbe-derived butyrate induces the differentiation of colonic Tregs by enhancing histone H3 acetylation in the promoter and conserved non-coding sequence regions of the Foxp3 locus and reduces the development of colitis [54]. Therefore, SCFAs may act as modulators of cancer and immune homeostasis.

A cell-based HDAC assay employed by Park et al. (2015), in which SCFAs must enter T cells to inhibit HDACs, found that the HDAC activity in T cells was dose-dependently suppressed by SCFAs, the HDAC activity of SCFAs was not diminished in T cells deficient in GPR41 or GPR43, and further studies showed that SCFAs regulate the mTOR-S6K pathway required for T-cell differentiation into effector and Tregs [38]. This suggests that SCFAs inhibit histone deacetylases in a GPR41/GPR43-independent manner and regulate the mTOR-S6K signaling pathway required for T-cell differentiation into effector and regulatory T cells, which play an important role in the regulation of tissue inflammation and immunity [51]. In a study using bone marrow-derived macrophages (BMDMs) as a model cell type, PV et al. (2014) found that n-butyrate treatment significantly reduced the levels of NO, IL-6 and IL-12p40 by inhibiting HDAC [55].

## 3. SCFA Regulation of Inflammation via GPCRs and HDAC 

**Figure 2:**
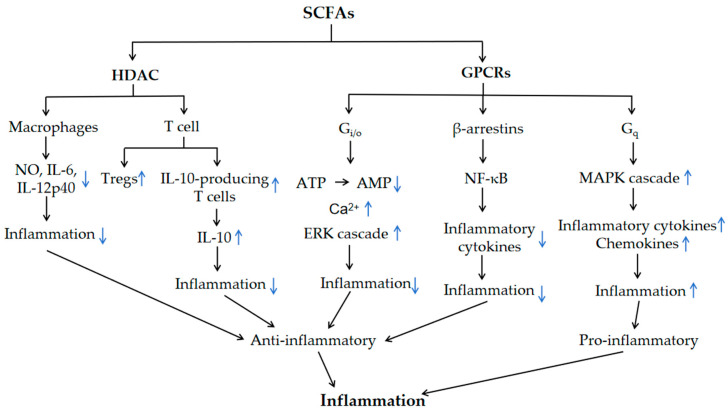
SCFAs regulates inflammation by acting on GPCRs and HDAC and regulating the production of cytokines in immune cells. GPCRs, G-protein coupled receptors; HDAC, histone deacetylase; ERK, signal-regulated kinase; MAPK, mitogen-activated protein kinase; NF-κB, nuclear factor-κB.

In immunity and inflammation, the recruitment and secretion of pro- and anti-inflammatory cytokines by immune cells plays a crucial role in protecting the organism from harm and maintaining the balance between the immune and inflammatory states [56]. However, when this balance is disturbed, as in the case of the excessive secretion of pro-inflammatory cytokines, systemic inflammation and pathological disease can result [57]. SCFAs modulate inflammation by regulating cytokine production in immune cells including neutrophils, macrophages, dendritic cells, T cells and B cells [56]. For example, SCFAs (butyrate and propionate) have been shown to reduce the expression of tumor necrosis factor (TNF) and nitric oxide synthase (NOS) in LPS-induced monocytes [58]. Macrophages are a major source of inflammatory mediators, and once activated, macrophages produce large amounts of TNF-α, IL-1β, IL-6, chemokines, nitric oxide (NO) and arachidonic acid derivatives [59]. SCFAs have been demonstrated to inhibit the production of pro-inflammatory mediators, including TNF-α, IL-6 and NO, which are stimulated by lipopolysaccharides (LPSs) and cytokines [59]. As illustrated in Figure 2, SCFAs regulate the inflammation by acting on GPCRs and HDAC.

SCFA–GPR43 signaling is one of the molecular pathways whereby commensal bacteria regulate immune and inflammatory responses [41]. In a study by KM et al. (2009), it was demonstrated that acetate induced apoptosis in neutrophils in a dose-dependent and GPR43-dependent manner [41]. Furthermore, acetate stimulation of human neutrophils markedly reduced the surface expression of pro-inflammatory receptors such as C5aR and CXCR2, presumably through agonist-mediated receptor heterodimerization and internalization [41]. It has been demonstrated that acetate/propionate can improve airway inflammation by acting on GPR41, reducing eosinophilic infiltration and downregulating the levels of IL-4, IL-5, IL-13 and IL-17A in the lungs [60]. Propionate ameliorates allergic airway inflammation and inhibits Th2 cytokine production by acting on GPR41 [61]. In addition to free fatty acid receptors, SCFAs have also been demonstrated to participate in the regulation of inflammation by binding to GPR109A [56]. GPR109A signaling induces anti-inflammatory properties in colonic antigen-presenting cells, which in turn promotes the differentiation of Tregs and IL-10-producing T cells [62]. Butyrate can induce the expression of anti-inflammatory molecules in macrophages and DCs through GPR109A signaling, thereby enabling them to support the differentiation of Tregs and IL-10-producing T cells [62]. Furthermore, GPR109A was found to be essential for the expression of IL-18 [62]. In a model of colitis induced by DSS in mice, Li et al. (2021) found that butyrate significantly inhibited IBD neutrophils from producing pro-inflammatory cytokines, chemokines and calprotectins, while the blockade of GPCR signaling with pertussis toxin (PTX) did not interfere with the effect; additionally, the HDAC inhibitor trichostatin A (TSA) efficiently mimicked the action of butyrate, suggesting that butyrate significantly ameliorates inflammation by inhibiting HDAC function to suppress the neutrophil-associated immune response such as the formation of pro-inflammatory mediators [27].

On the contrary, the pro-inflammatory effects of SCFAs have also been reported. In rat and in vitro experiments, SCFAs have been shown to increase the expression of the surface adhesion molecule L-selectin and the release of the cytokine CINC-2αβ (cytokine-induced neutrophil chemoattractant-2αβ), which in turn increases the migration of neutrophils to the site of inflammation, thereby exacerbating the inflammatory response [63]. SCFAs activate the MAPK pathways in enterocytes by activating GPR43, which in turn promotes the expression of the inflammatory cytokines IL-6, IL-17A and IL-12 and the chemokines CXCL1 and CXCL2 [29]. The two opposite effects of SCFAs, pro-inflammatory and anti-inflammatory, may be related to their local concentration, but the possible mechanisms still need to be further explored [56].

## 4. Physiological and Pathological Effects of SCFAs

### 4.1. Acetate

#### 4.1.1. Biosynthesis, Distribution and Physiology

Acetate production pathways are widely distributed in the microbiota. For example, acetate can be produced by enteric bacteria and acetogens (*Blautia hydrogenotrophica*) via the acetyl-CoA and Wood–Ljungdahl pathways, respectively [64]. Acetate and propionate are released into the portal vein, in addition to butyrate, which is consumed locally by colon cells. Acetate is the most abundant short chain fatty acid in both the colon and the peripheral circulation [3].

As the major SCFA produced by the gut microbiota, acetate can signal from the extracellular compartment to the cytoplasm by activating the protein-coupled receptor GPR43 [65]. Acetate can bind and activate GPR43 on neutrophils and eosinophils to induce their apoptosis, and drinking acetate-containing water can reduce inflammatory infiltration and tissue damage, resulting in a significant reduction in inflammation [29,41]. Acetate can also bind to liver GPR43 via the entero–liver axis and inhibit the carcinogenic IL-6/JAK1/STAT3 signaling pathway, thereby preventing the progression of non-alcoholic fatty liver disease-associated hepatocellular carcinoma (NAFLD-HCC) [66]. Furthermore, acetate can cross the blood–brain barrier and reduce appetite by changing the expression profiles of appetite regulatory neuropeptides in the hypothalamus through activation of the TCA cycle [67].

#### 4.1.2. Acetate and Diseases

In a rodent experimental model of colitis, oral administration of acetate has been shown to have a protective effect [42]. Acetate can reduce the levels of pro-inflammatory cytokines and modulate microglial phagocytosis and disease progression during neurodegeneration [14]. In a study involving transgenic mice overexpressing heparinase (Hpa-Tg mice), acetate was significantly decreased, and supplementation with acetate reduced neutrophil infiltration to alleviate acute pancreatic (AP) inflammation in Hpa-Tg mice [68]. Wang et al. (2020) found that Evodiamine (EVO) can regulate the keystone bacteria *L. acidophilus* to increase the production of acetate in the gut affected by colitis, thereby preventing or treating ulcerative colitis (UC) [69]. In a human epithelial cell culture model derived from colitis patients, high acetate administration was found to have protective effects on epithelial resistance, barrier gene expression and inflammatory protein production [70]. The production of acetate plays a crucial role in pulmonary immunity against pneumococcal infection [71]. The study demonstrated that acetate therapy augmented the bactericidal activity of alveolar macrophages by eliminating the NLRP3 inflammasome and glycolytic–HIF-1A axis in the context of Streptococcus pneumoniae, irrespective of either GPR43 or acetyl-CoA synthetases 1 and 2 [72]. Additionally, a proliferative probiotic Bifidobacterium strain in the gut can ameliorate the progression of metabolic disorders through microbiota modulation and acetate elevation [73]. A recent study showed that sodium acetate has a bidirectional regulatory effect on macrophages and can also affect lipid accumulation in hepatocytes [74]. Acetate has also been found to improve Canavan disease [75], prevent hypertension [76] and suppress non-alcoholic fatty liver disease [66]. As the major SCFA produced by the gut microbiota, the regulatory role of acetate in host metabolic control is important. However, there are many issues that require extensive work to fully understand the roles of acetate in host metabolic control and to provide better strategies for the prevention and treatment of human diseases.

### 4.2. Propionate

#### 4.2.1. Biosynthesis, Distribution and Physiology

Propionate is produced via the succinate, acrylate or propanediol pathways [14]. Propionate production is dominated by relatively few bacterial genera. *Akkermansia municiphilla* has been identified as a key propionate-producing species that degrades mucin [64]. Propionate is absorbed by the intestinal tract into the portal vein and metabolized in the liver, and it is only present at low concentrations in the peripheral circulation [3]. Despite the low peripheral concentration, propionate affects peripheral organs indirectly by activating the hormonal and nervous systems [8].

Propionate activates intestinal gluconeogenesis via the gut–brain neural circuit, thereby promoting metabolic benefits on body weight and glucose control [77]. As an endogenous HDAC2 inhibitor, propionate can decrease the levels of apoptosis of intestinal epithelial cells caused by oxidative stress [78]. Propionate has also been shown to promote colonic homeostasis and health [79]. Propionate protects from cardiac damage and reduces atherosclerosis in experimental hypertension, and its effects may depend on Tregs [80]. Lesley et al. (2018) found that propionate also has a beneficial protective effect on the blood–brain barrier, where the mechanism is due to inhibiting inflammation and oxidative stimulation [81].

#### 4.2.2. Propionate and Diseases

Propionate is a major microbial fermentation metabolite in the gut of many animals including humans, and it exerts versatile health-promoting effects systemically beyond the gut [82], including the attenuation of cardiac hypertrophy, fibrosis and vascular dysfunction [81] and the amelioration of colonic inflammation [83]. These studies suggest that propionate in the gut can modulate the pathogenesis of systemic diseases and gut-specific immune responses [61]. Propionate-producing bacteria play a critical role in the protection from inflammatory arthritis [84]. The level of propionate on the skin surface was significantly lower in atopic dermatitis (AD) patients than in healthy individuals, and topical application of propionate attenuated skin inflammation in mice with MC903-induced AD-like dermatitis by inhibiting IL-33 production in keratinocytes [85]. With both human and animal model studies, Arash et al. (2022) demonstrated that propionate attenuates atherosclerosis through the immune-dependent regulation of intestinal cholesterol metabolism [86]. Si et al. (2024) found that *Achyranthis bidentatae* could attenuate renal injury in STZ/HFD mice by modulating the abundance of beneficial flora, thereby promoting the production of propionic and isobutyric acids [87]. Propionate supplementation promotes the expansion of peripheral regulatory T cells in patients with end-stage renal disease (ESRD) [88]. The cardioprotective effect of propionate is also dependent on Tregs [80]. These studies highlight the immunomodulatory role of propionate and its importance for cardiovascular health.

### 4.3. Butyrate

#### 4.3.1. Biosynthesis, Distribution and Physiology

Butyrate can be produced via the phosphotransbutyrylase/butyrate kinase pathway and the butyryl-CoA:acetate CoA transferase pathway [12,89]. In addition, some microorganisms in the gut (*Anaerostipes* spp., *Coprococcus catus*, *Eubacterium rectale*, *Eubacterium hallii*, *Faecalibacterium prausnitzii*, *Roseburia* spp.) can also synthesize butyrate from lactate and acetate, which prevents the accumulation of lactic acid and stabilizes the intestinal environment [8]. Butyrate is the preferred energy source for colon cells [8], and it is estimated that up to 95% of microbial-produced butyrate is consumed by the colon, where it plays an important regulatory role in intestinal barrier function and inflammation [90]. There is also a small amount of butyrate transported through the intestine into the blood circulation, affecting the function and metabolism of peripheral tissues [3]. Despite the low peripheral concentrations, butyrate can indirectly affect physiological functions by activating hormones and the nervous system [8]. As a major product of gut microbial fermentation, butyrate has been recognized as an important mediator of gut microbiota regulation in whole body energy homeostasis [91]. Butyrate increases peroxisome proliferator-activated receptor-gamma coactivator 1α (PGC-1α) expression and adenosine monophosphate-activated kinase (AMPK) phosphorylation in muscle and liver tissue and PGC-1α and mitochondrial uncoupling protein-1 (UCP-1) expression in brown adipose tissue, promoting fatty acid oxidation and thermogenesis [92]. Like propionate, butyrate can also activate intestinal gluconeogenesis via the gut–brain neural circuit, thereby promoting metabolic benefits for weight and glucose control [77]. Significant changes in SCFAs, particularly the butyrate concentrations in blood or tissues, cause inflammatory, immunological and metabolic diseases [93]. Appropriate concentrations of butyrate help to maintain normal metabolism in the prevention and treatment of diseases [94]. In a limited number of investigations conducted to date, butyrate or sodium butyrate has been used therapeutically in diseases in vivo and in vitro. Research conducted on the application of butyrate in diseases is summarized in Table 3.

#### 4.3.2. Butyrate and Diseases

##### Obesity

The prevalence of overweight and obesity is increasing. It has been estimated by the Global Burden of Disease Obesity Collaborators that >107.7 million children and >603.7 million adults are obese [109]. Obesity and overweight are important determinants of a range of health problems and increase the risk of many related diseases including type 2 diabetes mellitus (T2DM), cardiovascular disease (CVD), non-alcoholic fatty liver disease (NAFLD), cognitive impairment and others [94]. The role of butyrate in obesity has been studied in humans, as well as in vitro and in vivo animal studies [110,111,112]. In HFD-induced obese mice, treatment with butyrate leads to improved energy metabolism via reducing energy intake and enhancing fat oxidation by activating brown adipose tissue (BAT) [113,114]. In mouse models of obesity, dietary supplementation with butyrate can prevent and treat diet-induced obesity and insulin resistance [19]. According to a study involving 205 women at 16 weeks gestation, the butyrate production capacity was decreased in obese pregnant women [115]. Another study found that the abundance of butyrate-producing bacteria was significantly decreased in non-alcoholic steatohepatitis (NASH) [116]. These findings suggest that butyrate plays an important role in the development of obesity.

##### Diabetes

Butyrate exhibits correlative beneficial effects in glucose homeostasis. A reduction in butyrate-producing bacteria has consistently been found in individuals with T2DM, as well as in those with prediabetes [117]. Transplantation of T2DM-susceptible bacteria can also reduce SCFA levels and GPR41/43 expression in rats [118]. In addition to its preventive effects on body weight and adiposity, butyrate supplementation has also been associated with the mitigation of insulin resistance in several animal models [110,119]. For example, sodium butyrate supplementation (5% wt/wt) into the HFD of C57BL/6J mice prevented HFD-induced adiposity and insulin resistance [120]. The pathogeneses of T2DM include glucose toxicity, oxidative stress, endoplasmic reticulum stress (ERS) and inflammation [97]. A study exposed mouse islets and INS-1E cells to a low dose of IL-1β and/or butyrate and measured inflammatory gene expression and nitric oxide (NO) production; the results showed that butyrate inhibited IL-1β-induced inflammatory gene expression and NO production by suppressing NF-κB activation and thereby possibly preserved beta cell function [121,122]. Hu et al. (2018) suggested that sodium butyrate protects islet cells from apoptosis through inhibiting the PERK-CHOP pathway of endoplasmic reticulum stress [97]. Jia et al. (2020) found that non-obese diabetic (NOD) model mice that were given butyrate by drinking water were protected against vancomycin-accelerated type 1 diabetes mellitus (T1DM) in maternal mice and their female offspring [123]. In addition, oral butyrate does not affect innate immunity and islet autoimmunity in individuals with long-standing T1DM [124].

Long-term elevated blood glucose levels can lead to diabetic complications such as cardiovascular disease, diabetic kidney disease and diabetic retinopathy [125]. Butyrate has been shown to have a beneficial effect on kidney disease in humans. Post-treatment of juvenile diabetic rats with butyrate showed that sodium butyrate treatment improved renal function and ameliorated the histological alterations, fibrosis, apoptosis and DNA damage in the kidney [126]. The in vitro and in vivo experiments showed that sodium butyrate can activate the kidney mitochondrial AMPK/PGC-1-α signaling pathway, thereby improving mitochondrial dysfunction in diabetic nephropathy (DN) [104]. Du et al. (2020) found that butyrate alleviated DN by mediating the miR-7a-5p/P311/TGF-β1 pathway [102]. Furthermore, butyrate provides a protective effect in db/db mice and HG/LPS-induced C2C12 myoblasts by suppressing autophagy and oxidative stress and activating the PI3K/AKT/mTOR pathway [50,101]. Sodium butyrate supplementation also ameliorates diabetic inflammation, diabetic retinopathy and diabetes-induced aortic endothelial dysfunction [98,99,100].

Taken together, these findings demonstrate the microbiota-regulating and diabetic therapeutic effects of butyrate, which can be used as a potential treatment for diabetes.

##### Inflammation

Butyrate is a particularly important SCFA with anti-inflammatory properties and is generally present at lower levels in inflammatory diseases associated with gut microbiota dysbiosis in mammals [127,128]. The principle mechanisms through which butyrate exerts its anti-inflammatory effects are the suppression of NF-κB activation, the inhibition of interferon γ production and the upregulation of peroxisome proliferator-activated receptor γ (PPAR γ), which may result from the inhibition of HDAC [129]. Oral administration of butyrate markedly ameliorated mucosal inflammation in DSS-induced murine colitis by inhibiting neutrophil-associated immune responses such as pro-inflammatory mediators and NET formation [27]. Sodium butyrate can effectively inhibit the inflammation of adipose tissue mediated by the NLRP3 pathway [105]. Butyrate attenuates lung inflammation by negatively modulating Th9 cells [130]. Sodium butyrate supplementation modulates the neuroinflammatory response aggravated by antibiotic treatment in a mouse model of binge-like ethanol drinking [131]. Patients with acute pancreatitis showed a decrease in butyrate producers compared to healthy subjects [132]. Butyrate supplementation shows a protective effect against inflammation.

##### Cancer

Butyrate provides energy for colorectal epithelial cells and inhibits inflammation and tumor formation [62]. Clostridium Butyricum: C. butyricum, which produces butyrate, inhibits proliferation and induces apoptosis in colorectal cancer (CRC) cells by interacting with the Wnt/β-catenin signaling pathway modulating the composition of the gut microbiota [133,134]. This decreases atherosclerosis and the secretion of secondary bile acids (BAs) closely related to cancer development, enhances the secretion of SCFAs and activates GPRs including GPR43 and GPR109A that suppress tumor progression [134]. Butyrate acts as a potent HDAC inhibitor in colon cancer cells, enabling cell cycle arrest, differentiation and apoptosis at physiological concentrations [135]. SIRT1, a member of the HDAC family, is known to be positively correlated with tumor growth [136]. In the study of the anti-tumor effect of butyrate on HCT116 colorectal cancer cells and its molecular mechanism, it was found that butyrate treatment inhibited cell proliferation and induced apoptosis by inhibiting the activation of the mTOR/S6K1 signaling pathway, partly via SIRT1 inhibition [137]. It is consistent with previous findings that the abundance of butyrate-producing bacteria and the rate of butyrate production are greatly diminished in the colon during ulcerative colitis and colon cancer [138,139]. Nagendra et al. (2014) found a tumor suppressor role for GPR109A-butyrate signaling in the colon, suggesting that commensals in the gut protect the host not only against colonic inflammation but also against colon cancer [62].

Recently, a study found that supplementation with a high-fiber diet and butyrate could significantly inhibit gastric cancer (GC) development, promote apoptosis of GC cells and inhibit their proliferation [140]. The study highlighted that the restoration of gut microbial butyrate enhanced CD8^+^ T-cell cytotoxicity via GPR109A/HOPX, thus inhibiting GC carcinogenesis, suggesting a novel theoretical basis for GC management [140]. In addition, another study found that butyrate induced ROS-mediated apoptosis by modulating the miR-22/SIRT-1 pathway in liver cancer cells [141]. Butyrate supplementation can also interfere with pancreatic cancer biology and the response to treatment and can alleviate some damage associated with cancer itself or to chemotherapy [142]. As an HDAC inhibitor, butyrate has great potential for future therapy against various cancers [143].

##### Kidney Diseases

An altered gut microbiota composition has been reported in patients with chronic kidney disease (CKD), including those with ESRD, compared with healthy controls [144,145]. Butyrate producers decreased with CKD severity [146]. Supplementation with *Faecalibacterium prausnitzii*, a beneficial butyrate-producing bacterium, reduced kidney dysfunction, kidney inflammation and the serum levels of various uremic toxins in CKD mice, and this was, at least in part, attributed to the butyrate-mediated GPR43 signaling in the kidney [147]. The mechanism by which butyrate improves kidney dysfunction remains unknown and thus requires further exploration [148]. In the adriamycin-induced nephropathy model, butyrate protects against proteinuric kidney disease through epigenetic- and GPR109A-mediated mechanisms [149]. In the kidney of rats subjected to contrast-induced nephropathy, sodium butyrate decreases the activation of NF-κB, thereby reducing inflammation and oxidative damage [150]. Wu et al. (2020) found that sodium butyrate may attenuate deoxycorticosterone acetate/salt-induced hypertension and renal damage by inhibiting the MR/SGK1 pathway [151]. The use of butyrate or butyrate-producing diets holds promise for the prevention and treatment of CKD. Gut dysbiosis has been reported in CKD, but the results of clinical trials testing probiotic supplementation were disappointing [147].

##### Neurological/Psychiatric Diseases

Accumulating evidence has shown that butyrate can reduce neurotoxicity, neuroinflammation and behavioral abnormalities, with benefits for a variety of central nervous system diseases through inhibiting HDAC [152,153]. Sodium butyrate prevents excessive ROS through NADPH oxidase 2 (NOX2) suppression and superoxide dismutase 1 (SOD1) upregulation through the p21/NRF2 (Nuclear factor erythroid 2-related factor 2) pathway in an HFD-induced obesity mouse model of Alzheimer’s disease (AD), which is critical for inhibiting beta-site amyloid precursor protein cleaving enzyme 1 (BACE1)-dependent amyloidogenesis in neuronal cells exposed to a high cholesterol environment [154]. Butyrate supplementation in mice improves neuroinflammation and mitochondrial impairment in the cerebral cortex and synaptic fraction in an animal model of diet-induced obesity [108]. Collectively, SCFAs influence the development of neurological disease by inhibiting histone acetylation, regulating the expression of tight junction proteins, or affecting microglial morphology and function [155].

### 4.4. Others

The primary source of SCFAs in humans is the metabolic by-products of complex starch fermentation in the gut, where commensal bacteria produce 1–6 carbon structures, mainly including acetate, propionate, butyrate, valerate and caproate [30]. Valerate and caproate are two kinds of short chain fatty acid subtypes produced by the gut microbiota [156], and unfortunately, valerate and caproate have not been studied as extensively as other SCFAs; thus, little is known about their physiological role.

According to a study involving 71 males and 29 females with DN, valerate and caproate concentrations decreased significantly in the advanced DN group, suggesting that lower serum levels of valerate and caproate predict the progression from DN to ESRD [156]. However, another study showed that the plasma valerate levels were significantly higher in patients with the primary or secondary outcome of coronary artery disease (CAD) or cardiovascular disease (CVD) than in those without CAD or CVD [157,158]. Zhang et al. (2024) found that valeric acid and isovaleric acid were significantly elevated in the serum of depressed patients compared to healthy people [159]. The association of the gut microbiome and its metabolites with different diseases appears to be diverse under different conditions, such as the host’s age, sex, genotype, diet and geography [157,158]. However, the concentration of SCFAs in healthy people is independent of age and sex [160]. This indicates that it is necessary to further investigate the variation in the gut microbiome and its metabolites in different diseases, so as to further clarify the association among SCFAs and diseases, and to establish a laboratory foundation for treating diseases based on the gut microbiota and its metabolites [156].

## 5. Conclusions and Outlook

**Figure 3:**
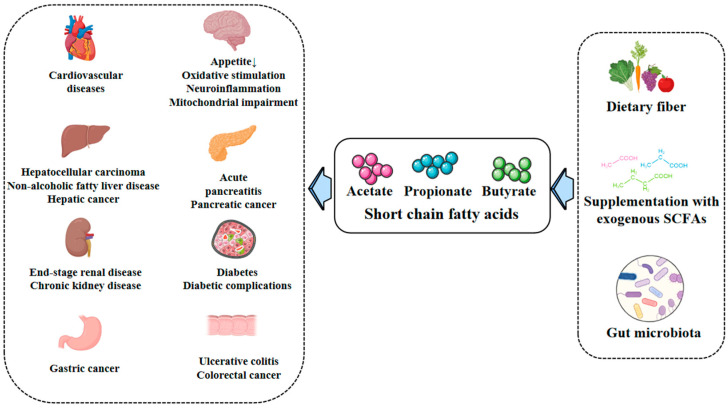
Gut microbial and supplementary sources of SCFAs regulate host metabolism, which is closely related to a variety of diseases.

As the major SCFAs produced by the gut microbiota, acetate, propionate and butyrate have attracted more and more attention from researchers, and a growing body of evidence supports SCFAs as key mediators that may help to prevent, reverse and delay the progression of diseases. Supplementation with endogenous or exogenous SCFAs improves body weight, glucose regulation, lipid metabolism and inflammation and is beneficial for a large number of people with metabolic diseases (Figure 3). As HDAC inhibitors, SCFAs can inhibit tumor cell proliferation, induce tumor cell apoptosis, inhibit inflammation and play important roles in the onset and development of cancer and inflammation. By activating GPCRs, SCFAs can reduce inflammation, improve islet metabolism and mitochondrial function and affect multiple tissues such as the cardiovascular system, pancreas and brain. In addition, it has been suggested that the activation of thermogenesis in brown adipose tissue and white adipose tissue may also be related to increased concentrations of SCFAs [161].

However, some of the mechanisms of SCFAs in physiology or pathology are still unclear. For example, in the inflammatory response, SCFAs exhibit both pro-inflammatory and anti-inflammatory opposing effects. The possible mechanisms are uncertain and remain to be further explored. In addition to acetate, propionate and butyrate, there are a few studies on valerate and caproate. Moreover, some studies have suggested that SCFAs may be harmful. A study found that increased acetate production due to a gut microbiota–nutrient interaction in high-fat-fed rodents leads to the activation of the parasympathetic nervous system resulting in increased ghrelin secretion and glucose-stimulated insulin secretion [162]. This generates a positive feedback loop resulting in hyperphagia, hypertriglyceridemia, ectopic lipid deposition in liver and skeletal muscle and insulin resistance in liver and muscle [162]. The tissue-specific effects of SCFAs have been demonstrated in the case of propionate, where propionate-dependent gluconeogenesis in the small intestine improves metabolic health, whereas hepatic gluconeogenesis is detrimental [8]. Administration of exogenous high-dose propionate in mice and humans results in rapid activation of the sympathetic nervous system, leading to increases in both glucagon and fatty acid-binding protein 4 [163]. The increases in both of these fasting hormones in the postprandial state drive enhanced endogenous glucose production, likely due to glycogenolysis, leading to hyperglycemia and compensatory hyperinsulinemia [163]. Thus, repeated daily exposure to propionate over prolonged periods may have important health implications. The effect of butyrate on glycolipid metabolism also remains controversial. Butyrate is capable of increasing lipid synthesis from acetyl-CoA or ketone bodies via the β-hydroxy-β-methylglutaryl-CoA pathway, which may contribute to obesity [164]. A small fraction of butyrate could be transported via the portal vein to the liver, where it is metabolized to yield acetyl-CoA and influences glycolipid metabolism [165]. As discussed above, the concentration of SCFAs plays an important role in the occurrence and development of the disease. Further basic experiments and well-designed clinical trials are needed to determine the mechanisms of SCFAs in the physiology and pathophysiology of the gut and other tissues or organs in the organism.

SCFAs are the major metabolite of the intestinal flora. More and more studies have shown that the composition of the intestinal flora is closely related to the occurrence and progression of various diseases. The disorder of the intestinal flora leads to a decrease in the fluctuation of biochemical factors and the contents of SCFAs, bile acids and endocrine-regulating peptides and, conversely, to an increase in the content of LPS, which then induces the occurrence of diseases [166]. On the contrary, a balanced intestinal flora could not only promote the body’s metabolism but also strengthen the body’s immune function [166]. Recent approaches to regulate the gut microbiota for the treatment of disease have mainly focused on probiotics, prebiotics, synthetics, fecal microbial transplantation, dietary interventions, bacteriophages, microbiota-targeted drugs and postbiotics. In addition, there is growing evidence that some natural herbal medicines can effectively improve disease by regulating the gut microbiota. As a commonly used natural herbal medicine in the clinical treatment of diabetes, mulberry leaves have been shown to regulate the composition of the intestinal flora, reverse the state of dysbiosis, increase the abundance of intestinal SCFA-producing bacteria and the levels of SCFAs in the intestinal tract, inhibit the level of inflammation in the body, regulate glucose–lipid metabolism and improve insulin secretion and insulin resistance [167]. Although common hypoglycemic drugs such as metformin are effective, they are prone to various adverse effects that make their use unsafe. Mulberry leaf, as a plant with the same origin as medicine and food, can ensure its safety while exerting its therapeutic effect. These treatments include natural herbal medicines and FMT, among others, which may be used in the future to regulate the intestinal flora and thus the concentration of SCFAs to improve the health of the organism. With the increasing awareness of the relationship between SCFAs and disease, we can hold high expectations that SCFA-related pharmacological agents may provide better strategies for health maintenance and disease prevention.

## Figures and Tables

**Table 2:** SCFA receptors and their main expression sites.

SCFA Receptors	G-Protein Coupling	Affinity	Expression	Ref
GPR43/FFAR2	G_i/o_; G_q_	C2 = C3 > C4 > C5 = C1 (human) C2 > C3 > C4 > C5 = C1 (mouse)	white adipocytes, enteroendocrine L cells, intestinal epithelial cells (IECs), pancreatic β-cells, colonic Tregs, M2 macrophages, neutrophils, eosinophils and mast cells	[13,22,31,33]
GPR41/FFAR3	G_i/o_	C3 = C4 > C5 > C2 > C1	sympathetic ganglion cells, enteroendocrine L cells, enteroendocrine K cells, white adipocytes, myeloid dendritic cells, thymus, pancreatic β-cells	[13,22,31,33]
GPR109A/HCA2	G_i/o_	C4, niacin	white adipocytes, brown adipocytes, keratinocytes, retinal-pigmented epithelium, immune cells (dermal dendritic cells, monocytes, macrophages, neutrophils)	[22,34,35,36]

C1, formate; C2, acetate; C3, propionate; C4, butyrate; C5, valerate.

**Table 3:** The effects of butyrate on diseases.

Disease	Model	Design	Results	Refs
Obesity	Female C57BL/6 mice on Western-style diet	5% (W/W) sodium butyrate supplementation in food for 12 weeks	BW ↓; TG ↓; liver weight ↓; Occludin ↑; plasma endotoxin ↓; GPR43 ↓; GPR41 ↑; GPR109A ↑	[95]
Type 1 diabetes	Female NOD mice	150 mM sodium butyrate in the drinking water for 36 weeks	BG ↓; Insulitis ↓; loss of insulin-positive cells ↓; serum C-peptide ↑; cTregs ↑; CXCL12 ↑	[96]
Type 2 diabetes	Male Sprague–Dawley rats on HFD	Pretreated with HFD for 4 weeks and one dose of STZ (35 mg/kg, IP), then sodium butyrate treatment at a dose of 500 mg/kg/day (IP) for 6 weeks	BG ↓; HOMA-IR values ↓; fasting serum insulin levels ↓; TC ↓; TG ↓; Bax ↓; cleaved caspase-3 ↓; caspase-12 ↓; Bcl-2 ↑; p-PERK ↓; CHOP ↓	[97]
Diabetes	STZ-induced male C57BL/6 mice	5% (W/W) sodium butyrate supplementation in diet for 20 weeks	3-NT ↓; 4-HNE ↓; VCAM-1 ↓; ICAM-1 ↓; NRF2 ↑; NQO1 ↑; t-NRF2 ↑; c-NRF2 ↑; HDAC ↓; ROS ↓; MDA ↓	[98]
Insulin resistance	Male C57BL/6J mice on HFD	5% (W/W) sodium butyrate supplementation in diet for 12 weeks	BW ↓; BG ↓; insulin ↓; IR ↓; AMPK ↑; PGC-1α ↑; p38 ↑; CPT1b ↑; COX-I ↑; PPAR-δ ↑; TG ↓; TC ↓	[19]
Diabetic inflammation	Male db/db mice on HFD	Orally treated with sodium butyrate (0.5 g/kg/day) for 5 weeks	BW ↓; BG ↓; blood Cr ↓; BUN ↓; WBC ↓; ALYs ↓; IL-1β ↓; MCP-1 ↓; TNF-α ↓; IL-8 ↑; ZO-1 ↑; ICAM-1 ↓; ROS ↓; MDA ↓	[99]
Diabetic retinopathy	STZ-induced male C57BL/6J mice	Daily gavage with sodium butyrate (500 mg/kg) for 12 weeks	BG (↓); ZO-1 ↑; Occludin ↑; SCFA ↑	[100]
Diabetic nephropathy	Male db/db mice	1% (W/W) sodium butyrate supplementation in diet for 12 weeks	IL-6 ↓; TNF-α ↓; IL-1β ↓; CRP ↓; Muc2 ↑; TJ proteins ↑; ZO-1 ↑; Occludin ↑; LC3II ↓; p62 ↑; ROS ↓; MDA ↓; p-PI3K ↑; p-Akt ↑; p-mTOR ↑; FFA2 ↑	[101]
Diabetic nephropathy	Male db/db mice	1% (W/W) sodium butyrate supplementation in diet for 12 weeks	CoIV ↓; PAI-1 ↓; α-SMA ↓; CTGF ↓; TGF-β1 ↑; P311 ↓	[102]
Diabetic nephropathy	STZ-induced male C57BL/6 mice	5% (W/W) sodium butyrate supplementation in diet for 20 weeks	UACR ↓; TGF-β1 ↓; CTGF ↓; PAI-1 ↓; BIP ↓; CHOP ↓; MDA ↓; iNOS ↓; 3-NT ↓; Ho1 ↑; Nqo1 ↑; n-NRF2 ↑; HDAC ↓	[103]
Diabetic nephropathy	Male db/db mice	Daily gavage with sodium butyrate (1000 mg/kg) for 12 weeks	BW ↓; BG ↓; BUN ↓; Ucr ↓; TG ↓; TC ↓; UAE ↓; glucose tolerance ↓; IR ↓; SREBP-1c ↓; FAS ↓; PPAR-γ ↑; CPT-1 ↑; PPARα ↑; ACOX1 ↑; cleaved caspase 3 ↓; Bax ↓; Bcl-2 ↑; PGC-1α ↑; p-AMPK ↑	[104]
Inflammation	Male db/db mice	Sodium butyrate (1.0 g/kg, IP) every other day for 6 weeks	BW ↓; Glucose ↓; EAT ↓; SAT ↓; IL-1 ↓; IL-6 ↓; TNF-α ↓; NLRP3 ↓	[105]
Non-alcoholic fatty liver disease	Male C57BL/6J mice on HFD	Daily gavage with sodium butyrate (200 mg/kg) for 8 weeks	BW ↓; ZO-1 ↑; MCP-1 ↓; TNF-α ↓; TGF-β1 ↓; α-SMA ↓; Smad7 ↓; Smad2 ↓; MCP-1 ↓; IL -1 ↓; IL -2 ↓; IL-6 ↓; IFN-γ ↓; TLR4 ↓; Myd88 ↓; IL-4 ↑; IL-10 ↑; PPAR-γ ↑	[106]
Non-alcoholic fatty liver disease	Male C57BL/6 J mice on HFD	Fed with HFD for 16 weeks and daily gavage with sodium butyrate (200 mg/kg) for the latter 8 weeks	BW ↓; IL-1β ↓; IL-6 ↓; TNF- α ↓; TC ↓; TG ↓; LDL-C ↓; HDL-C ↓; ALT ↓; AST ↓; CXCR4 ↓; miR-150 ↑	[107]
Neurological disease	Male C57BL/6J mice on HFD	Gavage with butyrate (100 mg/kg q.d.) for 6 weeks	BW ↓; TG ↓; CHOL ↓; leptin ↓; ADP ↓; TNF-α ↓; IL-1β ↓; IL-6 ↓; IL-10 ↑; ROS ↓; MDA ↓; GSH ↑; GSH/GSSG ↑; SOD ↑; Aconitase ↑; BDFF ↑	[108]

ACOX1, Acyl-CoA Oxidase 1; ADP, adiponectin; ALYs, abnormal lymphocytes; AMPK, AMP-activated protein kinase; Bax, Apoptosis Regulator; BIP, binding immunoglobulin protein; blood Cr, blood creatinine; BG, body glucose; BUN, urea nitrogen; BW, body weight; CHOL, cholesterol; CHOP, C/EBP homologous protein; CPT-1, Carnitine Palmitoyltransferase 1; CTGF, connective tissue growth factor; EAT, epididymal adipose tissue; FAS, Fatty acid synthase; GSH, glutathione; GSSG, oxidized glutathione; HDAC, histone deacetylase; HOMA-IR, homeostasis model assessment of insulin resistance; IL-6, interleukin-6; IL-1β, interleukin-1β; IL-8, interleukin-8; iNOS, inducible nitric oxide synthase; IR, insulin resistance; MCP-1, monocyte chemotactic protein-1; MDA, Malondialdehyde; Nqo1, dehydrogenase quinone; n-NRF2, nuclear NRF2; Ho1, Heme oxygenase 1; 3-NT, 3-Nitrotyrosine; PAI-1, plasminogen activator inhibitor-1; p-AMPK, phosphorylated AMP kinase; P311, an RNA-binding protein; PPARα, Peroxisome Proliferator Activated Receptor Alpha; PPAR-γ, Peroxisome Proliferator Activated Receptor Gamma; ROS, reactive oxygen species; SAT, subcutaneous adipose tissue; SCFA, short chain fatty acid; SOD, superoxide dismutase; SREBP-1c, Sterol-regulatory element binding proteins; STZ, streptozotocin; TC, total cholesterol; TG, triglyceride; TGF-β1, transforming growth factor-β1; TNF-α, tumor necrosis factor-α; UAE, urinary albumin; ZO-1, zona occludens-1.

## Data Availability

Not applicable.
